# Aquaglyceroporin-3’s Expression and Cellular Localization Is Differentially Modulated by Hypoxia in Prostate Cancer Cell Lines

**DOI:** 10.3390/cells10040838

**Published:** 2021-04-08

**Authors:** Andreia de Almeida, Dimitris Parthimos, Holly Dew, Oliver Smart, Marie Wiltshire, Rachel J. Errington

**Affiliations:** Tissue MicroEnvironment Group, Division of Cancer and Genetics, School of Medicine, Cardiff University, Cardiff CF14 4XN, UK; parthimos@cardiff.ac.uk (D.P.); hollye.dew@gmail.com (H.D.); oliversmart94@gmail.com (O.S.); wiltshirem@cardiff.ac.uk (M.W.); erringtonrj@cardiff.ac.uk (R.J.E.)

**Keywords:** aquaporins, aquaglyceroporin-3, prostate cancer, hypoxia, translocation

## Abstract

Aquaporins are required by cells to enable fast adaptation to volume and osmotic changes, as well as microenvironmental metabolic stimuli. Aquaglyceroporins play a crucial role in supplying cancer cells with glycerol for metabolic needs. Here, we show that AQP3 is differentially expressed in cells of a prostate cancer panel. AQP3 is located at the cell membrane and cytoplasm of LNCaP cell while being exclusively expressed in the cytoplasm of Du145 and PC3 cells. LNCaP cells show enhanced hypoxia growth; Du145 and PC3 cells display stress factors, indicating a crucial role for AQP3 at the plasma membrane in adaptation to hypoxia. Hypoxia, both acute and chronic affected AQP3′s cellular localization. These outcomes were validated using a machine learning classification approach of the three cell lines and of the six normoxic or hypoxic conditions. Classifiers trained on morphological features derived from cytoskeletal and nuclear labeling alongside corresponding texture features could uniquely identify each individual cell line and the corresponding hypoxia exposure. Cytoskeletal features were 70–90% accurate, while nuclear features allowed for 55–70% accuracy. Cellular texture features (73.9% accuracy) were a stronger predictor of the hypoxic load than the AQP3 distribution (60.3%).

## 1. Introduction

Aquaporins (AQPs) are cell membrane channels expressed in most tissues, with critical implications in the pathophysiological adaptation of single cells and cellular communities to the tissue microenvironment. Thus, these small proteins provide opportunities for therapeutic targeting [1,2,3].

From the thirteen mammalian AQPs, only channels in the aquaglyceroporin subfamily (AQP3, 7, 9, 10 and 11) are permeable to glycerol, water and other small non-charged molecules, such as hydrogen peroxide. AQP3 is the most ubiquitous glycerol channel, expressed mostly in the skin, where its function in glycerol permeation is responsible for skin hydration, elasticity and, therefore, important during wound healing [4]. This isoform is also expressed in various mucosa and epithelial tissues [5], reproductive organs [5] and is the only glycerol channel in human erythrocytes [6]. In addition to water and glycerol, AQP3 is permeated by hydrogen peroxide (H_2_O_2_), regulating downstream NOX-signaling [7], T-cell migration [8] and NF-kB signaling in keratinocytes and the development of psoriasis [9].

Several diseases have been correlated to AQPs, be that up/downregulation or complete loss of protein function. Specifically, the de-regulation of aquaglyceroporins has been shown to affect wound healing, fat metabolism, insulin resistance, cell proliferation and carcinogenesis [3]. Typically, AQP-driven responses to tissue perturbations include—fast cellular volume changes (ms), enhanced metabolic adaptation (s-min), vesicle release (min-h), and downstream protein translocation/expression (min-h) changes. AQPs present differently in various (patho) physiologies and change the set-point for mediating responses to match the altered microenvironment dynamics and the downstream effectors. Enhanced expression of aquaglyceroporin isoforms has been reported, among others, in colorectal, lung, gastric and human skin carcinomas [10] and linked with metastasis [11]. Glycerol is an essential metabolite in lipogenesis, glycolysis and gluconeogenesis, as well as an energy substrate to produce ATP via the mitochondrial oxidative phosphorylation, which may be of relevance in cells with high-energy demand such as cancer cells [12,13]. Tumors overexpressing glycerol channels are hypothesized to use glycerol as a pyruvate intermediate via the glycerol-3-phosphate (G-3-P) shuttle, leading to increased AQP production. However, other than the glycolytic pathway and ATP production, G-3-P can also be used in phospholipid biosynthesis, helping proliferating cells in growing cellular membranes.

AQP3 is expressed in both non-cancerous and cancerous human prostatic tissue [5,14,15,16,17]. Even though a few in vitro and in vivo experimental models have been used to elucidate the expression of AQP3 in the prostate, little is known about its function. AQP3 shows differential spatiotemporal patterns that alter in the prostate: such as relocation from plasma membrane expression in the prostate epithelium to intracellular compartments in prostate tumors [16]. Cytoplasmic rescue of AQP3 leads to its decreased availability for functional permeation at the membrane. Furthermore, AQP3 inhibition has been shown to prevent apoptotic characteristics in tumor cells by preventing apoptotic-related volume changes [18,19]. Thus, a working hypothesis is that AQP translocation to the cytoplasm may be the mechanism that enhances the anti-apoptotic setpoint in cells. On the other hand, a study by Bründl et al. showed a correlation between AQP3 expression and PSA levels and Gleason score [15].

While it is commonly assumed that AQPs are constitutively expressed in cell membranes, where they act as functional channels, recent studies have been pointing to a crucial role of AQP translocation between the cytoplasm and cellular compartments/vesicles into the plasma membrane [20]. Known triggers of translocation of glycerol channels include isoprenaline (AQP3, AQP7), adrenaline (AQP3), hypotonicity (AQP3) and hypertonicity (AQP3, AQP9) [20].

A characteristic feature of prostate tumors is their low oxygen levels [21]. Hypoxic regions develop within solid tumors as a result of accelerated oxygen consumption by rapidly proliferating cancer cells, in addition to a poorly formed vasculature [22]. The reduced oxygen levels reported in various tumor types relative to the healthy tissue are noteworthy, and the oxygen levels in the prostate are particularly low in healthy tissue. Prostate tumors have low oxygen levels (0.3–1.2%) when compared to normal prostate tissue (3.4–3.9%) [21,23]. Untreated LNCaP tumors have been shown to have as low as 0.8% oxygen levels [24]. Physiological levels of oxygen vary widely from 2–15%, depending on tissues [23]. “Normoxia” refers to atmospheric levels of oxygen and is commonly used in tissue culture conditions. Indeed, in this study, we have defined normoxia as 20% oxygen and hypoxia at 1%. Hypoxia has been associated with prostate tumor progression as it can allow for the selection of cells, which are androgen receptor(AR)-negative with abrogated apoptotic potential and a more invasive phenotype and being implicated in the epithelial to mesenchymal transition [24,25,26]. While AQP3 is upregulated in cancer cells, in response to low oxygen levels [27,28,29], healthy cells decrease their expression under hypoxia [30]. Hypoxia-inducible factors (HIFs) appear to induce AQP3 in cancer and, conversely, the aquaglyceroporin also stabilizes the hypoxic factors [31,32], the expression of the two is inversely correlated in healthy cells.

We took three different cell lines, each isolated from different metastatic sites, with different p53 status, and showed their different behavior in low oxygen conditions. We decipher these cell lines’ heterogeneity: it is a complex matrix of AQP3 localization in each cell line and the changes experienced in short, long, and after recovery to low oxygen (1%). We extended the approach to reveal the emergent features of the PC panel against a variable hypoxia burden.

## 2. Materials and Methods

### 2.1. Cell Culture

Prostate cancer cells (Du145, PC3 and LNCaP) and lung adenocarcinoma (A549) were obtained from ATCC (ATCC, Virginia, United States). All prostate cancer cell lines were cultured in RPMI-1640 (Gibco^TM^, Massachusetts, United States), and A549 cells were maintained in DMEM (high glucose, Gibco^TM^, Massachusetts, United States). All media was supplemented with 10% FBS (Sigma-Aldrich, Missouri, United States) and 1% penicillin/streptomycin (Gibco^TM^, Massachusetts, United States), and cells were cultured at 37 °C in a humidified atmosphere of 95% of air and 5% CO_2_ (Heraeus, Germany), unless otherwise specified.

For hypoxia (1% O_2_), cells were maintained at 37 °C in a humidified atmosphere of 95% of air and 5% CO_2_ (Heraeus, Germany). Cells were cultured under hypoxic conditions for either 5, 8 or 15 days (acute hypoxia) or 8 weeks (chronic hypoxia). For recovery, cells were cultured for 4 weeks under hypoxia and cultured for further 4 weeks under normoxia.

### 2.2. Flow Cytometry (AQP3 Expression and Cell Cycle)

For total AQP3 expression by flow cytometry, samples of each cell line were prepared from a 70–80% confluent culture and with a final cell number of 100,000 cells/sample. Cells were initially washed twice with 1× PBS (Gibco^TM^, Massachusetts, United States) and subsequently fixed with 4% paraformaldehyde (Sigma-Aldrich, Missouri, United States) for 30 min at room temperature (r.t.). Afterward, cells were incubated with 100 µL 1:200 dilution of anti-AQP3 antibody (rabbit anti-human, ab125219; Abcam, Cambridge, U.K.), in 1× PBS with 5% normal human serum (NHS, Invitrogen, Massachusetts, United States) and 0.1% Triton X-100 (PlusOne, Amersham Biosciences, Amersham, U.K.) at r.t., for 1 h. Staining with primary antibody was followed by incubation with 100 µL 1:500 secondary goat anti-rabbit Alexa Fluor^®^488 (ab150077, Abcam, Cambridge, U.K.), in 1× PBS with 5% NHS and 0.1% Triton X-100 also for 1 h and at r.t., in the dark. Cells were then washed and resuspended in 200 µL of 1× PBS. Before analysis, 2 µL (A549, Du145 and PC3) or 3 µL (LNCaP) of DRAQ5^TM^ (Biostatus, Leicestershire, U.K.) was added to each sample and incubated for 10 min, at 37 °C. Cells were kept on ice and away from direct light until analyzed using a BD FACSCalibur flow cytometer (BD Bioscience; California, United States). In each experiment, A549 cells in normoxia were analyzed and used as a control. To determine antibody specificity for AQP3, the corresponding blocking peptide (ab195690, Abcam, Cambridge, U.K.) was used according to the manufacturer’s instructions.

Results were analyzed using FlowJo 10.6.1, and the gating strategy is outlined in Figure S1. First, samples were gated to remove debris. Afterward, stained, unstained and blocked samples were compared to gate the positive population, and the mean fluorescence intensity (geometric mean) was taken from each positive sample peak. Both isotype control (rabbit IgG, ab171870 Abcam, Cambridge, U.K.) and a blocking peptide (ab195690, Abcam, Cambridge, U.K.) were used to ensure that the staining was AQP3-specific. Data for the AQP3 signal were normalized for the A549 normoxic samples within each experiment to decrease day-to-day variability in staining. Cells under normoxia were kept in parallel to the hypoxia incubations, and normoxia samples for all cell lines were analyzed various times throughout the 8 weeks incubations. This allowed us to ensure that any expression differences were not due to cell passage or other external factors.

Cell cycle data were acquired using DRAQ5^TM^ as a nucleic acid marker, and the gating strategy is shown in Figure S1. The cell cycle was divided into early and late phases. Here, individual cell cycle samples were not compared to normoxic A549 samples or normalized. For both AQP3 expression and cell cycle, results are shown as mean ± SEM of a minimum of three independent experiments.

### 2.3. mRNA Isolation and Quantitative RT–PCR

Extraction and isolation of mRNA was performed using the RNeasy mini kit (QIAGEN, Venlo, The Netherlands), using the manufacturer’s instructions. Afterward, reverse transcription (RT) was performed using a high-capacity cDNA reverse transcriptase kit (Applied Biosystems, Massachussets, United States), with a final volume of 20 μL per reaction, following the manufacturer’s instructions, using an Applied Biosystems “Gene Amp PCR System 2400” thermocycler (Applied Biosystems, Massachussets, United States),. The sample concentration used for RT was 1 µg of RNA in 10 µL of the sample (100 µg/mL). Two negative controls were used, one no-template control and another without reverse transcriptase, to assess reaction efficiency, using RNAse-free water (HyClone^TM^, Cytiva, Malborough, U.K.) as a substitute. Finally, quantitative PCR (qPCR) was carried out using 10 µL of TaqMan^TM^ gene expression master mix (20×) (Life Technologies), 4 µL of DNAse-free water (HyClone^TM^, Cytiva, Malborough, U.K.)), 1 µL of primer (primer concentration 50 µM) and 5 µL of cDNA. The cDNA sample was prepared by diluting the resulting RT sample in 80 µL of RNAse-free water (HyClone^TM^, Cytiva, Malborough, U.K.)) to a total of 100 µL. The two previous negative controls were used and an additional negative qPCR control, using DNAse-free water as a cDNA substitute. The following primers were used, all TaqMan^TM^ gene assays (Life Technologies, California, United States) with FAM reporter: AQP3—Hs00185020_m1, HIF-1α—Hs00153153_m1, GAPDH—Hs02758991_g1). Amplification was performed using a StepOne real-time PCR system thermocycler (Applied Biosystems, Massachussets, United States), using the following protocol: initial sample heating to 50 °C for 2 min, followed by heating at 95 °C for 10 min; repeating cycles of 95 °C for 15 s and 60 °C for 1 min, for a total of 40 cycles. Following data acquisition, baseline and thresholds were set using the StepOne software from Applied Biosystems (Applied Biosystems, Massachussets, United States). Data were further analyzed using the 2^−ΔΔCT^ method and are shown as mean ± SEM of three independent experiments.

### 2.4. Immunocytofluorescence and Fluorescence Microscopy

For the immunocytofluorescence assays, cells were seeded 24 h before the corresponding endpoint (5, 8 or 15 days, or 8 weeks). Different cell densities were used for the different cell lines to achieve 70–80% confluency at the endpoint and are as follows: Du145—16,000 cells/cm^2^, PC3—21,000 cells/cm^2^, LNCaP—32,000 cells/cm^2^. Cells were seeded at the densities described for each cell line on µ-Slide 8-well chambered polymer coverslip slides (Ibidi^®^, Germany). Afterward, cells were incubated for 24 h, either under 20% oxygen (normoxia) or 1% oxygen (hypoxia), 37 °C in a humidified atmosphere. Once cells were allowed to adhere for 24 h, media was removed, wells were washed with 1× PBS and subsequently fixed with 4% paraformaldehyde (Sigma-Aldrich; Missouri, United States) for 30 min at room temperature (r.t.). Afterward, cells were incubated with 100 µL 1:200 dilution of anti-AQP3 antibody (rabbit anti-human, ab125219 Abcam, Cambridge, U.K.), in 1× PBS with 5% normal human serum (NHS, Invitrogen, Massachusetts, United States) and 0.1% Triton X-100 (PlusOne, Amersham Biosciences, Amersham, U.K.) at r.t., for 1 h. Staining with primary antibody was followed by incubation with 100 µL 1:500 secondary goat anti-rabbit Alexa Fluor^®^488 (ab150077, Abcam, Cambridge, U.K.), in 1× PBS with 5% NHS and 0.1% Triton X-100, also for 1 h and at r.t., in the dark. To visualize the F-actin cytoskeleton, cells were incubated for 30 min at r.t, in the dark, with 200 μL Alexa Fluor™ 555 Phalloidin (A34055, Life Technologies, California, United States) diluted at 1:500 in a solution of 1× PBS. Cells were subsequently stained using 100 µL of NucBlue™ Fixed Cell ReadyProbes™ reagent (DAPI, Life Technologies, California, United States), diluted following the manufacturer’s instructions, in 1× PBS. Following a 5 min incubation at r.t., cells were imaged in the wells without washing or removing nuclear staining solution.

To account for unspecific staining, control staining comprised incubation with a rabbit immunoglobulin (IgG) isotype control (ab171870 Abcam, Cambridge, U.K.) in place of the primary antibody. However, due to the limitations of using an isotype control, and AQP3 blocking peptide (ab125219 Abcam, Cambridge, U.K.) was also used, which contains the epitope sequence of the AQP3 primary antibody. Cells were incubated for 30 min with 12.5 μL/mL of the AQP3 peptide (ab125219 Abcam, Cambridge, U.K.), representing an excess of 5× compared to the primary antibody concentration. This was followed by incubation with the AQP3 primary antibody and the Alexa Fluor^®^488 secondary antibody as described above.

Cells were imaged using a Zeiss LSM 880 confocal microscope (Zeiss, Germany) with Zen software, and images were processed for visualization using ImageJ version 2.0.0-rc-69/1.52. For or batch image analysis, images were acquired using a Zeiss Axiovert 100 (Zeiss, Germany) inverted fluorescence microscope, using MetaMorph^®^ software (Zeiss, Germany).

### 2.5. Image Analysis and Classification

CellProfiler version 3.1.8 2 (open-source) was employed for feature identification on images acquired with Zeiss Axiovert 100 inverted fluorescence microscope (Zeiss, Germany), as described above. Individual cells and nuclei were segmented following the methods and thresholds described in Table S1. Due to the cell’s unique features in the various conditions, different methodologies were used for the different cell lines and different conditions. Moreover, following the segmentation of cytoskeleton and nucleus, the latter was excluded from the whole cytoskeleton mask, generating a mask corresponding to the cell’s cytoplasm.

Shape features were subsequently extracted for the cytoskeleton and nucleus regions. Texture and AQP3 intensity features were obtained for the cytoplasm. Shape features include area, compactness, eccentricity, extent, form factor, major axis length, maximum Feret diameter, minimum Feret diameter, maximum radius, mean radius, median radius, minor axis length, orientation, perimeter and solidity. Texture measures obtained are angular second moment, contrast, correlation, variance, inverse difference moment, sum average of the normalized grayscale image in the spatial domain, the sum of the variance of the normalized grayscale image, sum entropy, entropy, difference of variance, a difference of entropy, infoMeas1, and infoMeas2. Due to differences in AQP3 signal between cell lines and conditions, we opted for selecting an appropriate exposure time per condition/cell line to better resolve the AQP3 localization to the detriment of resolving differences in intensity. Thus, from the intensity features, the only one used for this analysis was intensity mass displacement that, rather than measuring intensity, indicates where the higher intensity is, compared to the center of mass. Additionally, AQP3 distribution within the cytoplasm was further quantified within a set of four radially distinct areas (bins). The parameters obtained from this feature were fraction at a distance (FracAtD), the fraction of total stain in an object at a given radius; mean fraction (MeanFrac), mean fractional intensity at a given radius, and RadialCV, coefficient of variation of intensity within a ring. Images from this feature were saved. Of note, within this feature, each of the parameters is automatically normalized to the maximum intensity of each separate image. The FracAtD was selected as the parameter that best visually represents the intensity of the mass displacement parameter.

Machine learning classification was implemented on the MATLAB-based classification application suite (MATLAB R2020a, MathWorks, Massachussets, United States).

### 2.6. Statistical Analysis

Most statistical analyses were performed using GraphPad Prism 9 (California, United States). The following data were analyzed using one-way ANOVA, with Tukey’s multiple comparisons tests: flow cytometry AQP3 intensity, flow cytometry cell cycle distribution, qPCR results, imaging intensity mass displacement, cell area and nuclear area. Data from the cell cycle for intensity mass displacement (imaging) and AQP3 intensity (flow cytometry) were analyzed using two-way ANOVA with Sidak’s multiple comparisons test. Statistics involving machine learning were performed on MATLAB R2020a (MathWorks, Massachussets, United States). To confirm mathematical differences in the flow cytometry AQP3 intensity populations, prior to extracting parameters and further analysis, the population comparison tool from FlowJo^TM^ 10.6.1 was used. Representative examples are shown in Figure S2 and Table S2, showing the results for Cox chi-squared and Kolmogorov–Smirnov approaches, using 300 bins. All data are shown as mean ± SEM of at least three independent experiments.

## 3. Results

### 3.1. AQP3 Expression Levels under Normoxia and Hypoxia

**Figure 1:**
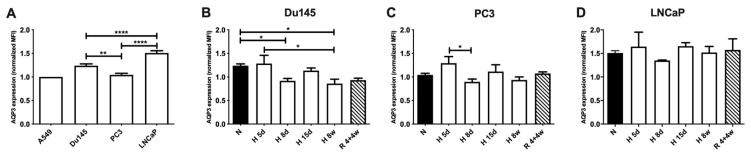
Total AQP3 protein expression in prostate cancer cells under normoxia (N—20% O_2_), hypoxia (H—1% O_2_) and recovery (R—hypoxia followed by normoxia). All data were normalized to the corresponding AQP3 expression in A549, under normoxia, within the same experiment. (**A**) AQP3 expression in all cell lines under normoxia. AQP3 expression for (**B**) Du145, (**C**) PC3 and (**D**) LNCaP cells under normoxia, acute hypoxia (5 d, 8 d, 15 d—days 5, 8 and 15), chronic hypoxia (8 w—8 weeks) and recovery (R 4 + 4 w—4 weeks hypoxia + 4 weeks normoxia). Data represent mean ± SEM of a minimum of three independent experiments. **** *p* < 0.0001 ** *p* < 0.01 * *p* < 0.05.

To assess the AQP3 expression levels in response to acute and longer-term exposure to hypoxia, total AQP3 protein expression was determined by flow cytometry following exposure to different oxygen levels and exposure times. Initially, to assess basal differences between cell lines, AQP3 expression was measured under normoxic conditions (20% O_2_), as shown in Figure 1A. All data were normalized to the expression levels of AQP3 under normoxic conditions of A549 cells as a control. While PC3 cells have the lowest basal levels, the highest AQP3 expression is observed for LNCaPs, and the trend for the studied cell lines is LNCaP > Du145 > PC3. This trend of expression appears to be inversely correlated to the metastatic potential of each cell line, with LNCaPs having the least metastatic potential of the panel and PC3 the highest [33].

After the initial evaluation of the protein expression levels between cell lines, cells were exposed to short-term (5, 8 and 15 days) or long-term (8 weeks) hypoxia (1% O_2_) (Figure 1B–D). To evaluate cells’ ability to recover from exposure to low oxygen, cells were exposed to 1% hypoxia for 4 weeks and normoxia for a following 4 week-period, “recovery”. Here, a pattern of AQP3 expression is observed for all cell lines: an initial increase at 5 days, followed by a decrease at 8 days and a subsequent increase at 15 days. Hypoxia expression levels were extremely similar between Du145 and PC3 cells, despite the significant difference in initial basal levels in normoxic conditions (Figure 1B,C). This led to a significant decrease in AQP3 expression when cells were exposed to long-term hypoxia or recovery in Du145 cells compared to normoxia, while the expression levels in PC3 cells are exposed to either 8 weeks hypoxia or recovery are similar to the normoxic levels. AQP3 expression in LNCaP cells showed little variation (Figure 1D), regardless of oxygen levels. In all cell lines, AQP3 expression for cells recovered from hypoxia is higher than those for hypoxia. Overall, it appeared that acute response to hypoxia resulted in a transient period with an initial increase in AQP3 expression, followed by a decrease. However, chronic hypoxia exposure lowered the AQP3 levels (Du145) or recovered to basal levels (PC3). Moreover, there was a trend for higher AQP3 expression for the recovery condition when compared to chronic hypoxia (not statistically significant). Therefore, we showed that AQP3 expression levels in recovered cells indicate previous exposure to hypoxic perturbations.

**Figure 2:**
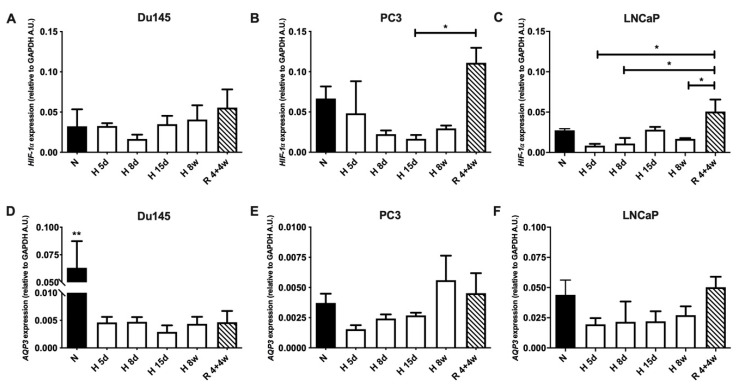
(**A**–**C**) Hypoxia-inducible factor (HIF)-1α and (**D**–**F**) AQP3 mRNA levels in prostate cancer under normoxia (N—20% O_2_), hypoxia (H—1% O_2_) and recovery (R—hypoxia followed by normoxia). HIF-1α mRNA expression for (**A**) Du145, (**B**) PC3 and (**C**) LNCaP. AQP3 mRNA expression for (**D**) Du145, (**E**) PC3 and (**F**) LNCaP. Data from cells under normoxia, acute hypoxia (5 d, 8 d, 15 d—days 5, 8 and 15), chronic hypoxia (8 w—8 weeks) and recovery (4 + 4 w—4 weeks hypoxia + 4 weeks normoxia). Levels of mRNA for both AQP3 and HIF-1α are relative to GAPDH levels for the same sample. Data represent mean ± SEM of a minimum of three independent experiments. ** *p* < 0.01 * *p* < 0.05.

We then investigated the mRNA levels of both hypoxia-inducible factor *HIF-1α* and *AQP3* in the PC cell panel under the same conditions (Figure 2). Regarding *HIF-1α* mRNA expression (Figure 2A–C), the pattern appeared more similar to that of AQP3 protein expression, which follows what has been described in the literature for cancer cells [31,32]. However, *HIF-1α* mRNA was lower than normoxia in most hypoxic conditions in all three cell lines. On the other hand, *HIF-1α* mRNA appeared the most elevated in the recovery conditions for all cell lines, significantly higher for both PC3 and LNCaP cells.

The *AQP3* mRNA levels in PC3 cells were 10-fold lower in normoxia than what was observed for both Du145 and LNCaP cells, despite a much smaller difference being observed at the protein level (Figure 2A–C). Additionally, *AQP3* levels in Du145 were higher than those of LNCaP cells, under normoxic conditions, despite the latter showing higher protein expression. Remarkably, even though the AQP3 mRNA of Du145 cells was comparable to that of LNCaP cells under normoxic conditions, once cells were exposed to hypoxia, these levels dropped 10-fold and were similar to those observed for PC3 cells. This was following what was observed for the protein expression where despite the normoxic AQP3 levels, Du145 and PC3 cells had similar protein expression levels when exposed to hypoxia, for both acute and chronic exposure. Both cell lines had a similar response regarding AQP3 protein and mRNA expression when exposed to low oxygen levels, regardless of their basal expression under normoxic conditions. For all cell lines, the *AQP3* mRNA did not appear to follow the same patterns as the protein expression but did appear to be lower under acute hypoxia.

### 3.2. AQP3 Expression Throughout the Cell Cycle

As bigger cells may appear brighter, when analyzing expression via flow cytometry, AQP3 intensity was analyzed against both forward (FSC) and side (SSC) scatter; no clear correlation was found for all three cell lines in all conditions. However, LNCaP cells are easily distinguishable from Du145/PC3 cells, based on their scatter properties, as shown in Figure S3. This property shows that they are smaller cells.

**Figure 3:**
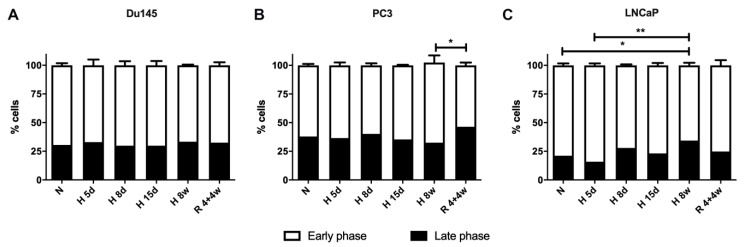
Cell cycle distribution in early or late phase for (**A**) Du145, (**B**) PC3 and (**C**) LNCaP cells, analyzed using flow cytometry. Data from cells under normoxia (N), acute hypoxia (5 d, 8 d, 15 d—days 5, 8 and 15), chronic hypoxia (8 w—8 weeks) and recovery (4 + 4 w—4 weeks hypoxia + 4 weeks normoxia). Data from (**A**–**C**) represent mean ± SEM of a minimum of three independent experiments. ** *p* < 0.01, * *p* < 0.05.

Cell cycle changes were also investigated under all the normoxic and hypoxic conditions for the PC panel and are shown in Figure 3. Initially, the cell cycle was analyzed from flow cytometry data, using an approach that classifies the cell cycle into early or late phases, as detailed in Figure S1. Interestingly, Du145 cells do not show any changed in the percentage of cells in the early or late phase of the cell cycle (Figure 3), regardless of oxygen levels. On the other hand, PC3 cells showed a significant increase in late phase cells in recovery when compared to long-term hypoxia, indicating that they might have grown faster after recovery from hypoxia. LNCaP cells appeared to go through more changes in the cell cycle than the other cell lines. In normoxia, the percentage of LNCaP cells in the late phase was lower than Du145 and PC3, consistent with the slower growth rate of LNCaPs (Figure S4). After 8 weeks of hypoxia exposure, more LNCaP cells in the late phase cell cycle were in agreement with a higher growth rate. Overall, cell cycle data were consistent with a maintained growth rate under hypoxia for PC3 and Du145 cells and an increased growth rate for LNCaP cells after chronic exposure. Notably, the cell line with the most stable AQP3 expression under hypoxia, LNCaP cells, was also the cell line with the highest cell cycle variation under the same conditions.

AQP3 expression is also tendentially higher in late phase cells in all cell lines (Figure S5). This is due to the nature of the late cell cycle when cells present a higher cytoplasm/nucleus ratio and have increased amounts of proteins.

Cell cycle data were also analyzed using microscopy data processed by CellProfiler [34]. DNA amount was estimated by using the total nuclear intensity for the nuclear label, multiplied by the nuclear area. Here, a similar approach was used to flow cytometry, and data were further processed using FlowJo^TM^, as detailed in Figure S6A–C. Here, early and late phase gating was used to further investigate if the cell cycle has any influence on AQP3 localization.

### 3.3. Cellular Distribution of AQP3

Aquaporins are membrane channels and are functional when inserted in cellular membranes, either organelles/vesicles or plasma membrane. We investigated the cellular localization of AQP3 in the PC cell panel.

**Figure 4:**
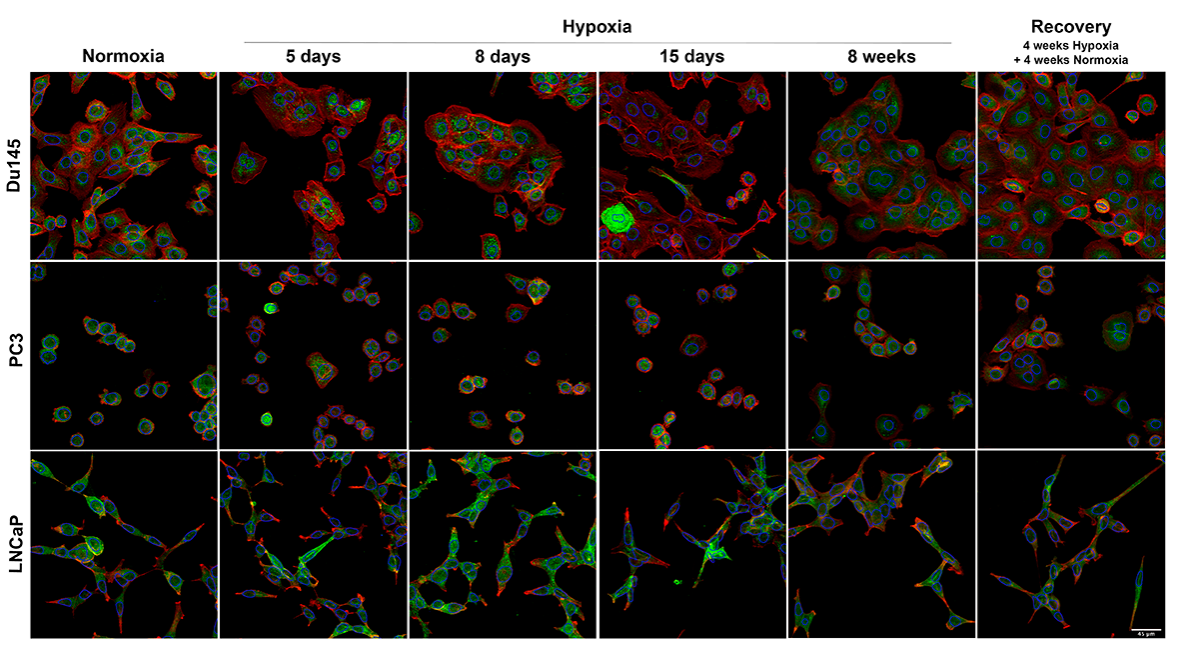
Maximum projection of Z-stack confocal microscopy images of Du145, PC3 and LNCaP cells under normoxia, acute hypoxia (days 5, 8 and 15), chronic hypoxia (8 weeks) and recovery (4 weeks hypoxia + 4 weeks normoxia). AQP3 is shown in green, while actin staining is shown in red. Nucleus is shown as a blue outline to allow clear observation of the green staining inside the nuclear area. Scale bar represents 45 µm.

**Figure 5:**
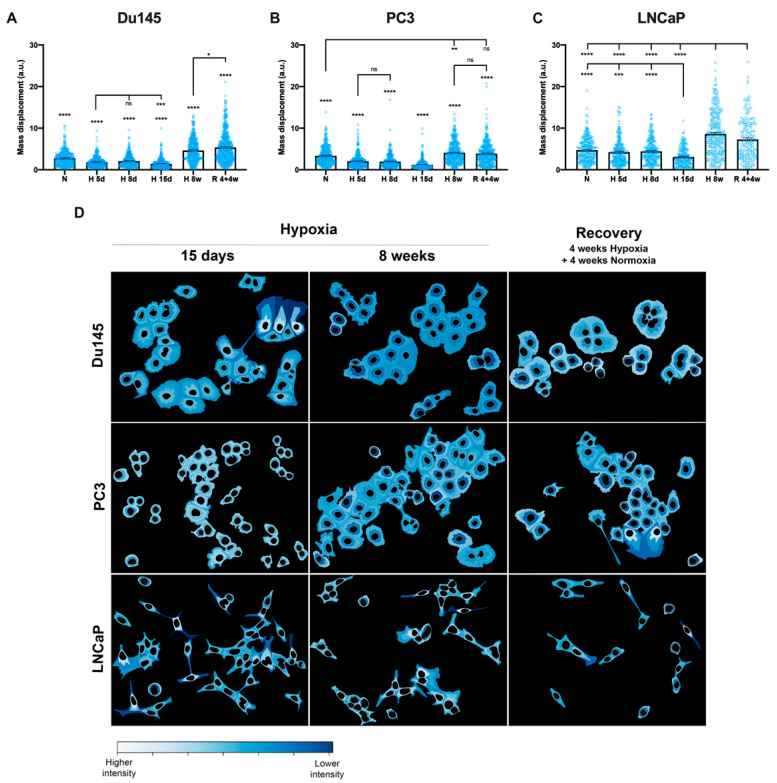
AQP3 cytoplasmic distribution. Mass displacement for AQP3 cytoplasmic labeling is shown for (**A**) Du145, (**B**) PC3 and (**C**) LNCaP cells. (**D**) Fraction of distance graphical representation of AQP3 intensity in the cytoplasmic compartment, using 4 bins. Scale of intensity is normalized for each image individually, for its maximum and minimum intensity, with a lighter color representing the highest intensity. Data from CellProfiler, from cells under acute hypoxia (5 d, 8 d, 15 d—days 5, 8 and 15), chronic hypoxia (8 w—8 weeks) and recovery (4 + 4 w—4 weeks hypoxia + 4 weeks normoxia). Each condition has, on average, 278 (Du145), 319 (PC3) and 217 (LNCaP) individual cells and data are shown as mean ± SEM. **** *p* < 0.0001, *** *p* < 0.001, ** *p* < 0.01, * *p* < 0.05, ns—not significant.

Here, we looked for variability in the cellular distribution of AQP3 in the different cell lines subjected to low oxygen. Remarkably, both the expression levels and the cellular localization of AQP3 were altered under acute and chronic hypoxia (Figure 4 and Figure 5). LNCaP cells were the only cells in the panel that show consistent AQP3 expression at the plasma membrane (Figure 4); AQP3 was mostly expressed at the cell membrane, with some cytoplasmic localization. Du145 and PC3 cells showed little AQP3 expression at the plasma membrane as most of its expressions were cytoplasmic (Figure 4). However, the expression pattern of cellular localization varied when cells were exposed to hypoxia. In an acute response, AQP3 expression decreased in the cytoplasm and increased in the perinuclear area. AQP3 staining had a very punctate pattern in all cell lines, but this was more apparent in Du145 cells.

After chronic exposure to hypoxia, AQP3 localization in Du145 cells was more homogeneously distributed but remained punctate in the cytoplasm. These cells also showed vacuole-like bodies in the cytoplasm. AQP3 staining was often observed lining these vacuolar structures and perinuclear (Figure S7). After the cells were allowed to recover from hypoxia for 4 weeks, the vacuolar bodies were no longer observed, indicating that these may have been caused by hypoxic stress. PC3 cells also showed vacuole-like structures, similar to Du145s, with AQP3 lining these structures.

To quantify the patterns of AQP3 protein localization, many images were acquired and further analyzed using CellProfiler [34]. The mass displacement parameter was used to quantify AQP3 distribution in the cytoplasm. In summary, after identification of the two cellular components (nucleus and whole cell), the nucleus was subtracted from the whole for each cell, generating a shape corresponding to the cytoplasm. The mass displacement index (MDI—a.u. units) indicates the distance of AQP3 labeling from the center of mass in each cell, with the highest index level (mean for maximum value ca. 9) indicating proximity to the cell membrane and the lowest index level (mean for minimum value ca. 1) indicating proximity to the nuclear border. As shown in Figure 5A–C, AQP3 labeling is proximal to the nucleus in acute (5–15 days) hypoxia for Du145 and PC3 cells (mean MDI ca. 1–2), which then in chronic (at 8 weeks) hypoxia conditions displayed a heterogeneous mass displacement index away from the perinuclear localization (mean MDI ca. 4–5) and was maintained in recovery (mean MDI ca. 4–5). Interestingly, differences were also observed in LNCaP cells, with AQP3 localization consistently proximal to the plasma membrane (mean MDI for normoxia and acute hypoxia ca. 3–5) and more heterogeneous in chronic hypoxia and recovery (mean MDI ca. 7–9). In all cell lines at 15 days, AQP3 labeling was observed the closest to the nuclear border (MDI Du145 1.40 ± 0.06, PC3 1.19 ± 0.06, LNCaP 3.08 ± 0.16). As shown in Figure S6H–J, the AQP3 intensity mass displacement, as a measure for AQP3 localization, was not cell cycle-driven.

A way of visualizing the mass displacement per cell can be shown by defining three zones, starting from the nuclear border (data are shown in Figure 5D and Figure S8). Here, it was possible to observe that at 15 days of hypoxia, AQP3 labeling was the most located (lightest color) closer to the nucleus, while it was located at the cell membrane and more heterogeneous for 8 weeks hypoxia and after 4 weeks recovery. In fact, in both Du145 and LNCaP cells, the mass displacement pattern in the recovery treatment conditions always remained unique compared to normoxia conditions only—AQP3 remains proximal to the cell membrane, which was significantly different from normoxia.

### 3.4. Classification of Cell Lines and Experimental Conditions

To unify an all-feature approach, we determined the morphological adaptations alongside biomarker expression under hypoxic stress. In the previous sections, we established the statistical significance of these apparent trends in several cases. However, a systematic comparison of multiple features requires multidimensional analysis. We advanced this by employing an array of machine learning tools that include decision trees, discriminant analysis, support vector machines (SVM), k-nearest neighbors, along ensemble combinations of these classifiers. Linear and medium Gaussian SVM generally outperformed other classifiers. These methodologies were applied to cytoskeleton, nucleus, AQP3 intensity, distribution and texture of labeling, obtained from CellProfiler [34].

Results are summarized in Table 1, making the case of cytoskeletal and nuclear shape features as predictors for environmental consequences. Overall classification accuracy (i.e., ability to distinguish the three cell lines for the six treatment regimens) was markedly higher for cytoskeletal rather than nuclear features. This finding agrees with the observation that nuclear shapes and sizes are fairly robust across the perturbations. The highest classification accuracy was obtained from the 15 days samples (88.3%), followed by 5 day samples (82.8%). The accuracy dropped at the 8 weeks case (72.7%) and even further at the 4 weeks hypoxia + 4 weeks recovery (69.4%). For specific cell lines, PC3 and LNCaP were identified with the highest accuracy (up to >90%), while DU145 identification was less accurate. Confusion matrices and ROC curves for Table 1 entries are included in the Supplementary Material (Figures S9–S13).

Employing AQP3 labeling texture features as predictors allowed classifiers to identify Du145 and PC3 cell lines with good accuracy but performed poorly on the LNCaP (Table 2). Cellular patterns determined at 15 days of hypoxia supported the highest classification accuracy, consistently with shape features presented in Table 1.

To identify the subset of specific features that account for the bulk of the strongly associated with classification accuracy (summarized in Table 1), we grouped the fifteen cytoskeleton-based predictors into six subgroups of associated features. These are: eccentricity + form factor (A), solidity + extent + orientation + compactness (B), max/min Feret diameter (C), major/minor axis length (D), max/min/mean radius (E), area + perimeter (F). Overall classification accuracy across the three cell lines (including all experimental conditions) for these subgroups was: 58.2% (A), 60.3% (B), 60.8% (C), 61.7% (D), 65.3% (E), 70.6% (F). Results suggest that size measurements (F) were the strongest predictors for the characterization of the three cell lines. Size information was also partially encoded in the max/min/mean radius (E), which achieved the second-best accuracy. Interestingly, in spite of the lowest overall accuracy, eccentricity + form factor (A) was able to strongly identify the LNCaP cell line with ~80% accuracy, which was consistent with the elongated shape of the cell. Combinations of the six feature groups show a graded increase in classification accuracy: 69.5% (D) + (E), 70.5% (D) + (E) + (C), 72.0% (D) + (E) + (C) + (F), and 72.9% for all fifteen predictors.

## 4. Discussion

Despite being functional channels in the cell membrane, it is not uncommon to find aquaporins located in other cellular compartments. AQP3 was shown to be located in the nucleus, cytoplasm and membrane of ER-silenced breast cancer cells and shown to be translocated from the nucleus into newly formed blebs [35]. Similarly, both AQP3 and AQP7 are found in both the nucleus and cytoplasm of hepatocellular carcinoma [36]. In PC, two previous studies have shown cytoplasmic AQP3 expression in cells and tissues while showing AQP3 at the membrane of healthy prostate cells [16]. Aquaporins were shown to translocate between different cellular compartments following different triggers [20]. AQP3′s translocation to the cell membrane can be triggered by a hormone, adrenaline [37], while spatiotemporal expression in ovaries is regulated by gonadotropin and steroid hormones [38]. Interestingly, one of the major differences between Du145/PC3 and LNCaP cells was the fact the former do not express the androgen receptor (AR), while the latter was androgen sensitive. Estrogen receptor (ER)-positive breast cancer cells have been shown to have higher AQP3 expression than ER-negative cells [39]. Moreover, the promoter of the *AQP3* gene was found to have an estrogen response element (ERE), which, once activated, could promote cell migration and invasion in these cells. Since LNCaP cells were the only AR-positive cells in the panel, the higher AQP3 expression and mechanism might be similar to ER-positive breast cancer cells. In fact, AQP3 has been identified as one of the genes that are upregulated (8.8) by androgen (dihydrotestosterone—DHT) in PC [40].

In ER-positive cells, AQP3 is only present at the cell membrane in higher histopathological grades and stage [39]; while these breast cancer cells have higher AQP3 expression and cell membrane localization, increasing tumor hypoxia also increases their growth rate and invasion [41]. This indicates that AQP3′s cellular localization at the plasma membrane may be playing a crucial role in the cellular adaptation to hypoxia, as seen in our study, for LNCaP cells. Hypoxia is known to enhance lipogenesis by using HIFs as modulators of various proteins involved in fatty acid uptake, metabolism and storage [42]. These include the PPARγ transcription factor, which promotes the uptake of extracellular fatty acids and is also a known regulator of AQP3 expression and shuttling [43,44]. Thus, AQP3 is an important player in hypoxia, regulating glycerol uptake as a fatty acid precursor to be used in lipogenesis. Glycerol can be an important player in oxidative phosphorylation (OxPhos) through the glycerol-3-phosphate (GG3P) shuttle, generating FADH_2_ [3]. By having AQP3 constitutively expressed at the cell membrane under normoxia, LNCaPs have an advantage under hypoxia, with faster adaptation and increased growth rate. On the other hand, Du145 and PC3 cells, which do not express AQP3 at the cell membrane under any of the studied conditions, display stress markers under chronic hypoxia. Higgins et al. have reported that LNCaP cells have significantly increased rates of OxPhos and lower rates of glycolysis than Du145 and PC3 cells under normoxic conditions [45]. OxPhos contributed 88% in LNCaPs, to the total ATP demand, while only 47% and 56% in Du145 and PC3 cells, respectively, which appeared to be mainly due to impaired mitochondria in Du145/PC3. This indicates that LNCaP cells may require glycerol as a crucial player in OxPhos, and therefore, constitutively express AQP3 at the cell membrane to cope with metabolic demand, while Du145/PC3 cells are not able to use glycerol in the same way due to impaired mitochondrial metabolism. Additionally, Higgins et al. showed that hypoxia significantly increased the glycolytic rate of LNCaP cells and not of Du145/PC3 [45]. An increased glycolytic rate would mean less demand for glycerol and less need for AQP3 to be localized at the cell membrane, explaining the cytoplasmic localization of AQP3 in Du145/PC3. In this study, we observe that AQP3 protein expression is altered both in protein levels and localization, while *AQP3* mRNA levels remain unaltered, with the exception of Du145 under hypoxia, when compared to normoxia. It is more advantageous for cells to regulate post-translationally AQP3 channels, as these are required by cells for fast volume and metabolism adaptation. Having basal AQP3 protein levels stored in the cytoplasm would mean that the cells can quickly adapt to microenvironmental stimuli by shuttling the channels to and from the cell membrane while requiring new protein to be expressed by altering *AQP3* mRNA levels would be a lengthier process. Here, we observed shuttling of AQP3 protein from closer to the cell membrane to closer to the perinuclear area in all cell lines, under acute hypoxia (up to 15 days), with a more heterogeneous localization under chronic hypoxia (8 weeks), remaining heterogeneous and closer to the cell membrane after reoxygenation. This pattern indicates that the cells are able to shuttle AQP3 from and to the plasma membrane (or closer to the plasma membrane, in the case of Du145/PC3) in response to metabolic adaptation under different levels of hypoxia. Moreover, the fact that LNCaP cells constitutively express AQP3 at the cell membrane appears to be advantageous, offering them a fast way to adapt and switch between OxPhos and glycolysis. Here we also showed that these differences in AQP3 expression and localization and not cell-cycle-driven.

In this study, we showed that *HIF-1α* mRNA levels are lower under hypoxia than normoxia and, in fact, several studies have found lower HIF-1α mRNA and protein levels under hypoxia after the first few hours of exposure [46,47,48]. On the other hand, *HIF-1α* mRNA is significantly higher in recovery conditions. An increased expression of the hypoxic factor in the recovery condition could be explained by the reoxygenation, mimicking angiogenesis and new blood supply to a tumor. In fact, cyclic (or intermittent) hypoxia, characterized by cyclic periods of hypoxia and reoxygenation, has been shown to increase the HIF-1α expression, stability and activity with each cycle [49]. We also observed that *HIF-1α* mRNA is lower for LNCaP cells under normoxia compared to the other cell lines, and this is consistent with what has been reported in the literature [45].

We also show that Du145 and PC3 cells have vacuole-like bodies when exposed to chronic hypoxia, and these are not present when cells are reoxygenated. Autophagy is a common cellular process through which cells digest damaged cellular components and is usually upregulated by stress factors [50]. Hypoxia promotes autophagy in solid tumors, and this process can have a tumor-suppressive role or enhance tumorigenesis [51]. PC3 cells show autophagy under hypoxia (for up to 48 h), characterized by the presence of lysosomal bodies and mediated by HIFs [52]. Additionally, both PC3 and LNCaP cells become more sensitive to hypoxia, with increased cell death, when autophagy is blocked [53]. The vesicular structures observed in chronic hypoxia for Du145 and PC3 cells can be a result of hypoxia-induced autophagy. Interestingly, AQP3 knockdown inhibits autophagy in gastric cancer cells [54]. Thus, AQP3 lining these structures might indicate that the glycerol channel is involved in the autophagy process in these cells by, among others, help the volume regulation of these vesicular bodies.

Employing machine-learning tools, we were able to identify unique features associated with individual cell lines and the various hypoxic conditions. Overall, PC3 and LNCaP cells have more cytoskeleton-unique features, which allow for more accurate classification. The highest classification accuracy between conditions was achieved through extracted cytoskeleton features, together with AQP3 intensity mass displacement providing predictors, as high as 93.5% for individual cell lines. Regarding different conditions, the highest accuracy was observed for 15 days of hypoxia, which is driven predominantly by the intensity mass displacement index. In fact, after 15 days, AQP3 is observed the furthest from the cell membrane in all cell lines. While LNCaP and PC3 cells have a unique shape, Du145 have similar features to PC3 cells, especially under certain hypoxic conditions, leading to a decreased classification accuracy for this cell line. Cells undergo cytoskeleton remodeling under hypoxia via various pathways. However, the effects of hypoxia on the cytoskeleton are highly cell-specific [55]. Hence, in the current study, cytoskeleton predictors in conjunction with AQP3 localization are able to accurately identify hypoxic exposure.

Regarding texture features, Du145 and PC3 cells have the highest overall accuracy of 70.2% and 73.9%, respectively, while it is lower for LNCaP cells, at 51.0%. The lower accuracy for LNCaP cells is linked to the fact that AQP3 is located at the cell membrane in all conditions. On the other hand, Du145 and PC3 cells alter not only their localization of AQP3 but also the expression pattern, and some conditions show more punctate patterns. The highest classification accuracy for Du145 cells was observed for recovery. In fact, in recovery, the pattern of AQP3 labeling is the most unique, and machine-learning was able to identify this. Here, cells display a punctate pattern and localization that is spread throughout the cytoplasm and is different from what is observed for both hypoxia and normoxia. In the case of PC3 cells, the highest classification accuracy was found for 15 days, which appears to be linked to shape features and how the AQP3 pattern is observed for cells of different shapes/flat surface areas.

Prostate tumors can present treatment resistance, which has been strongly linked to hypoxic tumor levels [21]. Using AQP3 as a spatiotemporal biomarker for hypoxic burden would allow patient stratification according to historical hypoxic exposure. This would improve outcomes and allow for better treatment design. Moreover, one of the main advantages of the proposed machine-learning methodology is its ability to perform classification into a multiparametric space. This multidimensional space can include phenotypical as well as genomic and proteomic quantifiers, thus generating a global biomarker signature. This approach is presenting one of the most promising techniques for advancing precision medicine.

## 5. Conclusions

The principal purpose of the current study was to quantify and map the changes in AQP3 expression in the three prostate cell lines under a critical environmental change, namely long and short-term changes in oxygen levels. There are very few studies that detect and measure potential biomarkers after chronic and adaptive exposure to low oxygen. Our work suggests that the spatiotemporal AQP3 expression could be used as a fingerprint of hypoxic burden/experience when comparing control with treated tissue. Finally, we have used cell painting [56] and AI methodologies to make our measurements and validate this, which is a similar approach taken by the phenotyping screeners, and so the foundational assay could be directly ported into a target screening pipeline [57].

Further work should focus on determining a combination of the morphology hallmarks and biomarker expression in patient biopsy material to predict and stratify hypoxic burden in tumors.

## Figures and Tables

**Table 1:** Machine-learning classification of Du145, PC3 and LNCaP cell lines based on 15 cytoskeletal and 15 nuclear geometric features (predictors). The different conditions are normoxia (N), acute hypoxia (5 d, 8 d, 15 d—days 5, 8 and 15), chronic hypoxia (8 w—8 weeks) and recovery (4 + 4 w—4 weeks hypoxia + 4 weeks normoxia). Overall classification accuracy and true-positive accuracy of individual cell lines are presented. Highest accuracy is shown in bold. MD—AQP3 mass displacement.

Condition	Feature	Overall Classification Accuracy (%)	Classification Accuracy per Cell Line (%)		
			Du145	PC3	LNCaP
N	MD + cytoskeleton predictors	75.5	65.0	88.0	69.0
	MD + nucleus predictors	62.3	37.6	75.6	68.7
H 5 d	MD + cytoskeleton predictors	82.8	75.1	85.7	87.8
	MD + nucleus predictors	69.6	60.7	76.4	70.1
H 8 d	MD + cytoskeleton predictors	75.1	63.2	74.9	**90.5**
	MD + nucleus predictors	57.9	39.2	47.6	64.3
H 15 d	MD + cytoskeleton predictors	**88.3**	**81.4**	**93.5**	86.6
	MD + nucleus predictors	65.6	47.3	81.2	58.1
H 8 w	MD + cytoskeleton predictors	72.7	52.6	75.8	88.5
	MD + nucleus predictors	61.6	38.7	72.0	70.9
R 4 + 4 w	MD + cytoskeleton predictors	69.4	77.4	52.0	81.1
	MD + nucleus predictors	55.7	51.4	43.1	38.4

**Table 2:** Machine-learning classification of individual experimental timepoints within each of the three cell lines (Du145, PC3, LNCaP). The different conditions are normoxia (N), acute hypoxia (5 d, 8 d, 15 d—days 5, 8 and 15), chronic hypoxia (8 w—8 weeks) and recovery (4 + 4 w—4 weeks hypoxia + 4 weeks normoxia). Classification was performed using texture cytoskeletal features and aquaporin distribution within the cytoskeleton as predictors. Highest accuracy is shown in bold.

Cell Line	Feature	Overall Classification Accuracy (%)	Classification Accuracy per Time Course (%)					
			N	H 5 d	H 8 d	H 15 d	H 8 w	R 4 + 4 w
Du145	Texture	70.2	**75.2**	63.4	57.6	73.0	69.2	**77.8**
PC3		**73.9**	74	**68.8**	**61.5**	**83.1**	**77.0**	75.6
LNCaP		51.0	61.4	43.3	34.8	59.2	58.6	45.9

## Data Availability

Data is contained within the article or Supplementary Materials. The corresponding authors can provide raw data of results upon reasonable request.

## Supplementary Materials

The following are available online at https://www.mdpi.com/article/10.3390/cells10040838/s1, Figure S1: Representative gating strategy for flow cytometry; Table S1: Parameters used in CellProfiler, for each cell line and condition; Table S2: Population comparison analysis from FlowJowTM; Figure S2: Population comparison analysis from FlowJowTM; Figure S3: Bubble plot of AQP3 expression, as normalised MFI, against (A) FSC or (B) SSC; Figure S4: Doubling times (h) for Du145, PC3 and LNCaP cells, when exposed to normoxia (20% oxygen), hypoxia (1% oxygen) and recovery (normoxia, followed by hypoxia); Figure S5: Cell cycle distribution of AQP3 expression by flow cytometry for (A) Du145, (B) PC3 and (C) LNCaP cells, analysed using flow cytometry; Figure S6: Cell cycle distribution from fluorescence microscopy data; Figure S7: Maximum projection of Z-stack confocal microscopy images of Du145 cells under chronic hypoxia (8 weeks), in greyscale and merged channels; Figure S8: Fraction at distance graphical representation of AQP3 intensity in the cytoplasmic compartment, using 4 bins; Figure S9: Confusion matrices summarising classification results for the three cell lines (DU145, LNCaP and PC3) when employing all six experimental conditions; Figure S10: Confusion matrix for all conditions, classifying the different cell lines and using a subset of cytoskeleton-derived intensity predictors, as listed; Figure S11: Confusion matrices for Normoxia, hypoxia (H) 5 days (5d) and 8 days (8d), 8 weeks (8w), and recovery (R 4+4w) used in the classification of DU145, LNCaP, PC3 cell lines; Figure S12: Confusion matrices for individual cell line, classifying the different conditions based on texture features predictor; Figure S13: Confusion matrix for all conditions, classifying the different cell lines based on texture feature predictors.
