# Supplementary material for: Aquaglyceroporin-3’s Expression and Cellular Localization Is Differentially Modulated by Hypoxia in Prostate Cancer Cell Lines
Source: Cells. 2021 Apr 8;10(4):838. doi: 10.3390/cells10040838 (PMC8068192; doi:10.3390/cells10040838)
Supplement: Supplementary file 1 [file cells-10-00838-s001.pdf]

Supplementary Material

Aquaglyceroporin-3's expression and cellular localisation is differentially modulated by hypoxia in prostate cancer cell lines

Andreia de Almeida [1*], Dimitris Parthimos [1], Holly Dew [1], Oliver Smart [1], Marie Wiltshire [1] and Rachel J. Errington [1]

[1] Tissue MicroEnvironment Group, Division of Cancer and Genetics, School of Medicine, Cardiff University, Cardiff CF14 4XN, UK

* Correspondence: deAlmeidaA@cardiff.ac.uk; Tel.: +44 (0)2920 687 342

Keywords: aquaporins; aquaglyceroporin-3; prostate cancer; hypoxia, translocation

---

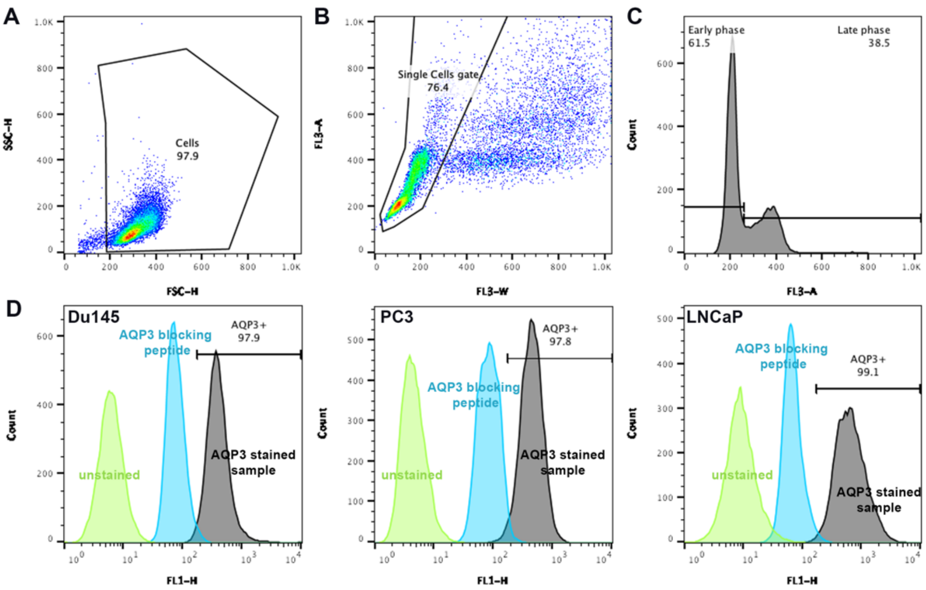

Figure S1: Representative gating strategy for flow cytometry. Representative sample of Du145 cells under normoxia, (A) gated to exclude cell debris. This gate was then used to further (B) gate for single cells, which resulting population was used to determine cell cycle (C) and AQP3 signal (D). Panel D shows a representative signal for AQP3 (grey) for the three prostate cell lines, as well as the signal for unstained controls and for samples with AQP3 blocking peptide, to determine antibody signal specificity. Data from FlowJow™

Table S1: Parameters used in CellProfiler, for each cell line and condition. Other parameters not mentioned were left as default.

Cell line	Condition	Identify primary objects (Nuclei)		Identify secondary objects (Cytoskeleton) Input objects: nuclei	
Du145	N	Obj. diam.: 20-85<br>Global, Otsu, Two classes	Threshold smooth. scale: 1.5<br>Threshold corr. factor: 0.9	Propagation, Global, Otsu, three classes, Background	Threshold smooth. scale: 1<br>Threshold corr. factor: 0.60
	H 5d	Obj. diam.: 20-85<br>Global, Otsu, Two classes	Threshold smooth. scale: 1.5<br>Threshold corr. factor: 0.9	Propagation, Global, Otsu, three classes, Background	Threshold smooth. scale: 1<br>Threshold corr. factor: 0.60
	H 8d	Obj. diam.: 10-85<br>Global, Otsu, Two classes	Threshold smooth. scale: 1.5<br>Threshold corr. factor: 0.9	Propagation, Global, Otsu, three classes, Background	Threshold smooth. scale: 1<br>Threshold corr. factor: 0.60
	H 15d	Obj. diam.: 10-65<br>Global, Otsu, Two classes	Threshold smooth. scale: 1.5<br>Threshold corr. factor: 0.9	Propagation, Global, Otsu, three classes, Background	Threshold smooth. scale: 1<br>Threshold corr. factor: 0.8
	H 8w	Obj. diam.: 10-80<br>Global, Otsu, Two classes	Threshold smooth. scale: 1.5<br>Threshold corr. factor: 0.9	Propagation, Global, Otsu, three classes, Background	Threshold smooth. scale: 1<br>Threshold corr. factor: 0.55
	R 4+4w	Obj. diam.: 10-80<br>Global, Otsu, Two classes	Threshold smooth. scale: 1.5<br>Threshold corr. factor: 0.9	Propagation, Global, Otsu, three classes, Background	Threshold smooth. scale: 1<br>Threshold corr. factor: 0.55
PC3	N	Obj. diam.: 20-80<br>Global, Otsu, Two classes	Threshold smooth. scale: 1.5<br>Threshold corr. factor: 0.9	Propagation, Global, Otsu, three classes, Background	Threshold smooth. scale: 1<br>Threshold corr. factor: 0.60
	H 5d	Obj. diam.: 20-80<br>Global, Otsu, Two classes	Threshold smooth. scale: 1.5<br>Threshold corr. factor: 0.9	Propagation, Global, Otsu, three classes, Background	Threshold smooth. scale: 1<br>Threshold corr. factor: 0.65
	H 8d	Obj. diam.: 20-80<br>Global, Otsu, Two classes	Threshold smooth. scale: 1.5<br>Threshold corr. factor: 0.9	Propagation, Global, Otsu, three classes, Background	Threshold smooth. scale: 1<br>Threshold corr. factor: 0.6
	H 15d	Obj. diam.: 1-70<br>Global, Otsu, Two classes	Threshold smooth. scale: 1.5<br>Threshold corr. factor: 0.9	Propagation, Global, Otsu, three classes, Background	Threshold smooth. scale: 1<br>Threshold corr. factor: 0.75
	H 8w	Obj. diam.: 1-70<br>Global, Otsu, Two classes	Threshold smooth. scale: 1.5<br>Threshold corr. factor: 0.9	Propagation, Global, Otsu, three classes, Background	Threshold smooth. scale: 1<br>Threshold corr. factor: 0.55
	R 4+4w	Obj. diam.: 20-75<br>Global, Otsu, Two classes	Threshold smooth. scale: 1.5<br>Threshold corr. factor: 0.75	Propagation, Global, Otsu, three classes, Background	Threshold smooth. scale: 1<br>Threshold corr. factor: 0.55
LNCaP	N	Obj. diam.: 30-75<br>Global, Otsu, Two classes	Threshold smooth. scale: 1.5<br>Threshold corr. factor: 0.75	Propagation, Global, Otsu, three classes, Background	Threshold smooth. scale: 1<br>Threshold corr. factor: 0.6
	H 5d	Obj. diam.: 30-65<br>Global, Otsu, Two classes	Threshold smooth. scale: 1.5<br>Threshold corr. factor: 0.75	Watershed Gradient, Global, Min cross entropy	Threshold smooth. scale: 0<br>Threshold corr. factor: 1
	H 8d	Obj. diam.: 30-70<br>Global, Otsu, Two classes	Threshold smooth. scale: 1.5<br>Threshold corr. factor: 0.9	Watershed Gradient, Global, Min cross entropy	Threshold smooth. scale: 0Threshold corr. factor: 1
	H 15d	Obj. diam.: 30-72<br>Global, Otsu, Two classes	Threshold smooth. scale: 1.5<br>Threshold corr. factor: 0.9	Watershed Gradient, Global, Min cross entropy	Threshold smooth. scale: 0<br>Threshold corr. factor: 1
	H 8w	Obj. diam.: 30-72<br>Global, Otsu, Two classes	Threshold smooth. scale: 1.5<br>Threshold corr. factor: 0.9	Watershed Gradient, Global, Min cross entropy	Threshold smooth. scale: 0<br>Threshold corr. factor: 1
	R 4+4w	Obj. diam.: 30-72<br>Global, Otsu, Two classes	Threshold smooth. scale: 1.5<br>Threshold corr. factor: 0.9	Watershed Gradient, Global, Min cross entropy	Threshold smooth. scale: 0<br>Threshold corr. factor: 1

Table S2: Population comparison analysis from FlowJowTM. Analyses were performed for one control normoxia sample or 8d H, against a combination of two control normoxia samples. Results are shown for Cox Chi Square and Kolmogorov-Smirnov approaches, using 300 bins.

Comparison	Chi-Squared T(X)	K-S Max Difference (%)	K-S Max at Intensity	K-S Probability
N *vs* N controls	58.3452	11.8	365.1741	>99.9%
8d H *vs* N controls	801.7444	47	294.2727	>99.9%

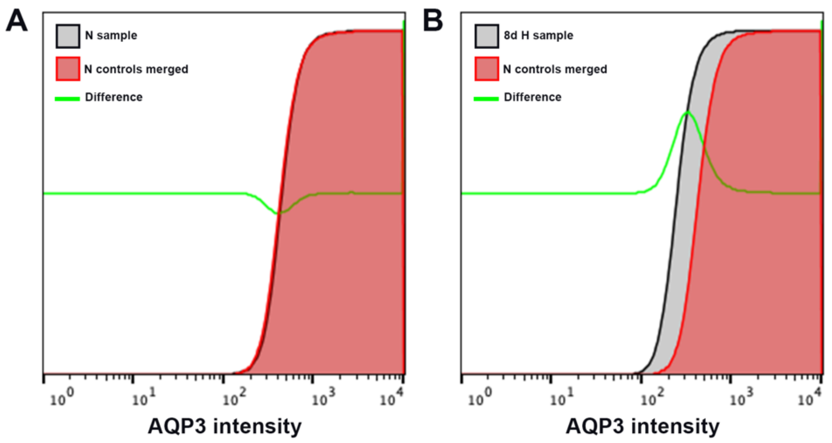

Figure S2: Population comparison analysis from FlowJow™. Analyses were performed for one control normoxia sample **(A)** or 8d H **(B)**, against a combination of two control normoxia samples. Data for AQP3 fluorescence intensity are shown as cumulative distribution function (CDF) for sample (grey) or control. Calculated difference between sample and control is shown as a green line. Results were calculated using 300 bins.

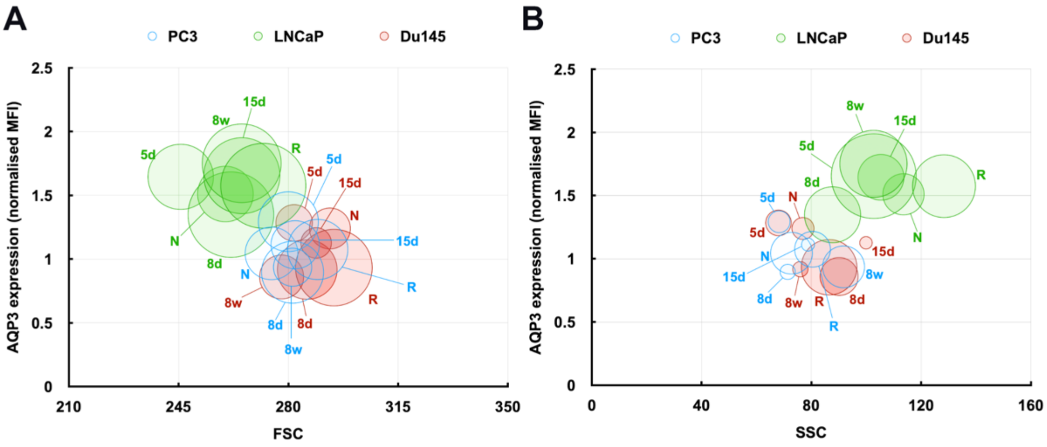

Figure S3: Bubble plot of AQP3 expression, as normalised MFI, against (**A**) FSC or (**B**) SSC. Data from cells under normoxia, acute hypoxia (5d, 8d, 15d - days 5, 8 and 15), chronic hypoxia (8w - 8 weeks) and recovery (4+4w - 4 weeks hypoxia + 4 weeks normoxia). Data represent mean ± SEM of a minimum of three independent experiments, where the bubble size is shown as SEM for FSC or SSC.

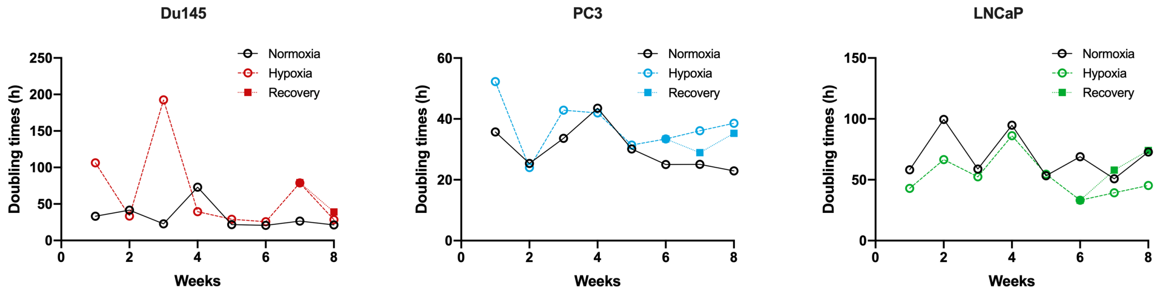

Figure S4 – Doubling times (h) for Du145, PC3 and LNCaP cells, when exposed to normoxia (20% oxygen), hypoxia (1% oxygen) and recovery (normoxia, followed by hypoxia).

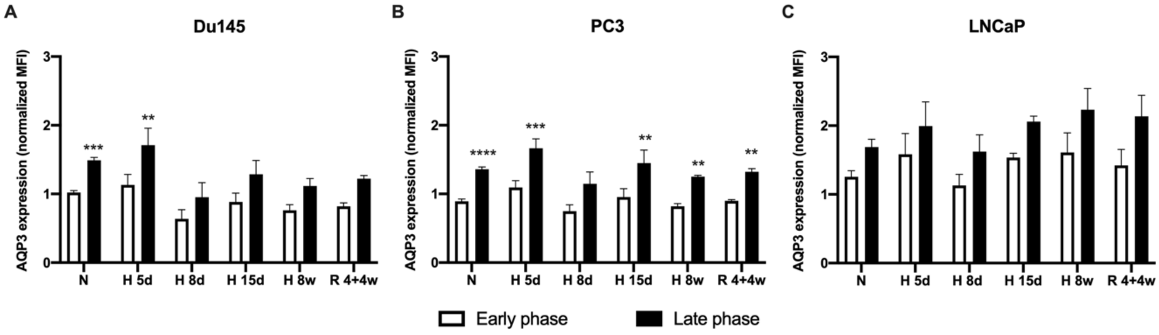

Figure S5: Cell cycle distribution of AQP3 expression by flow cytometry for (A) Du145, (B) PC3 and (C) LNCaP cells, analysed using flow cytometry. Data from cells under normoxia, acute hypoxia (5d, 8d, 15d - days 5, 8 and 15), chronic hypoxia (8w - 8 weeks) and recovery (4+4w - 4 weeks hypoxia + 4 weeks normoxia). Data from (A-C) represent mean ± SEM of a minimum of three independent experiments. ****$p<0.0001$, ***$p<0.001$, **$p<0.01$.

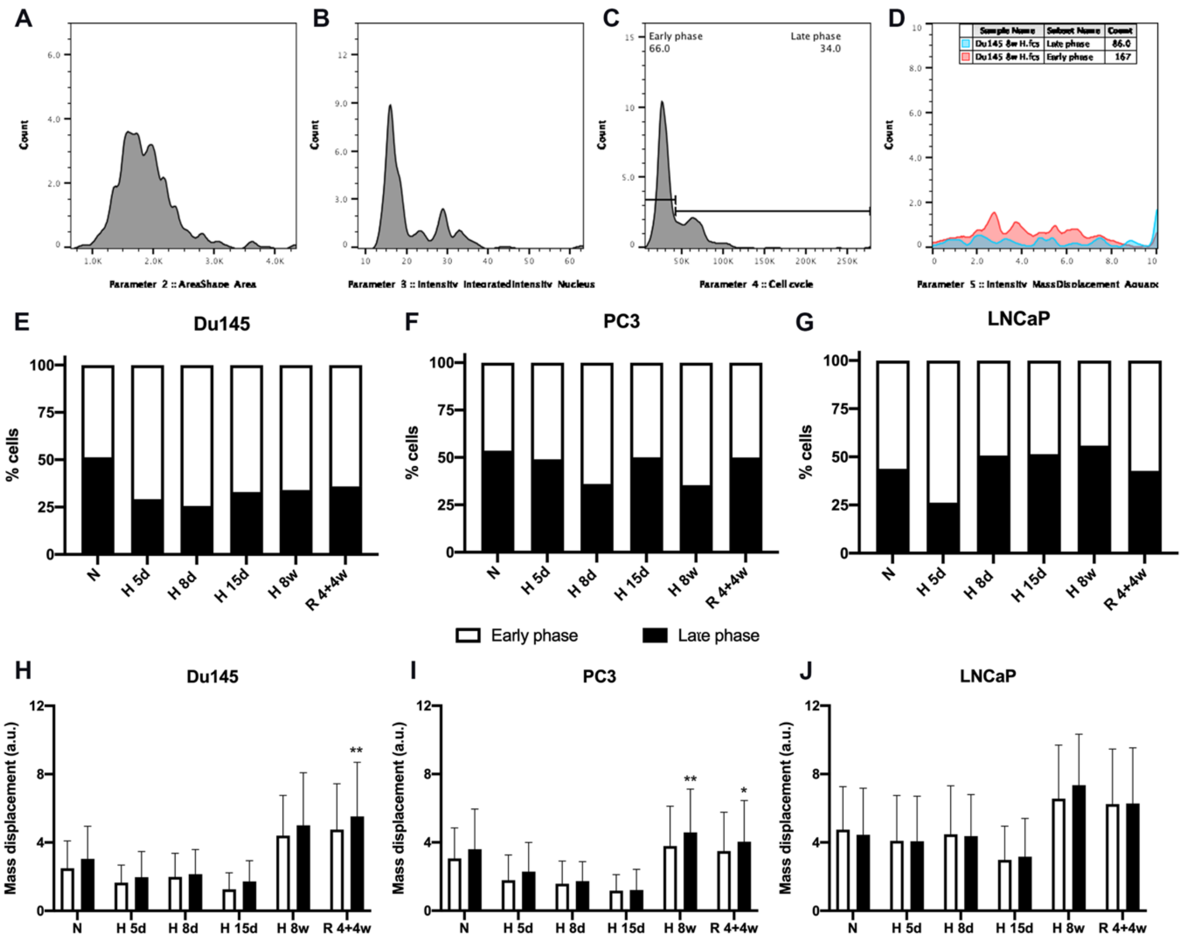

Figure S6: Cell cycle distribution from fluorescence microscopy data. Cell cycle data, as DNA content, were calculated as Area x Total nuclear intensity (**A-B**), and the gating strategy is shown in (**C-D**). Cell cycle distribution (**E-G**) is shown as percentage of cells in early or late phases of the cell cycle, as shown in (**C**). Mass displacement (**H-J**) for AQP3 staining is shown for early/late cell cycle phases. Data from CellProfiler, from cells under normoxia, acute hypoxia (5d, 8d, 15d - days 5, 8 and 15), chronic hypoxia (8w - 8 weeks) and recovery (4+4w - 4 weeks hypoxia + 4 weeks normoxia). Each condition has, in average, 278 (Du145), 319 (PC3) and 217 (LNCaP) individual cells and data for (**H-J**) are shown as mean and CV. **p<0.01, *p<0.05

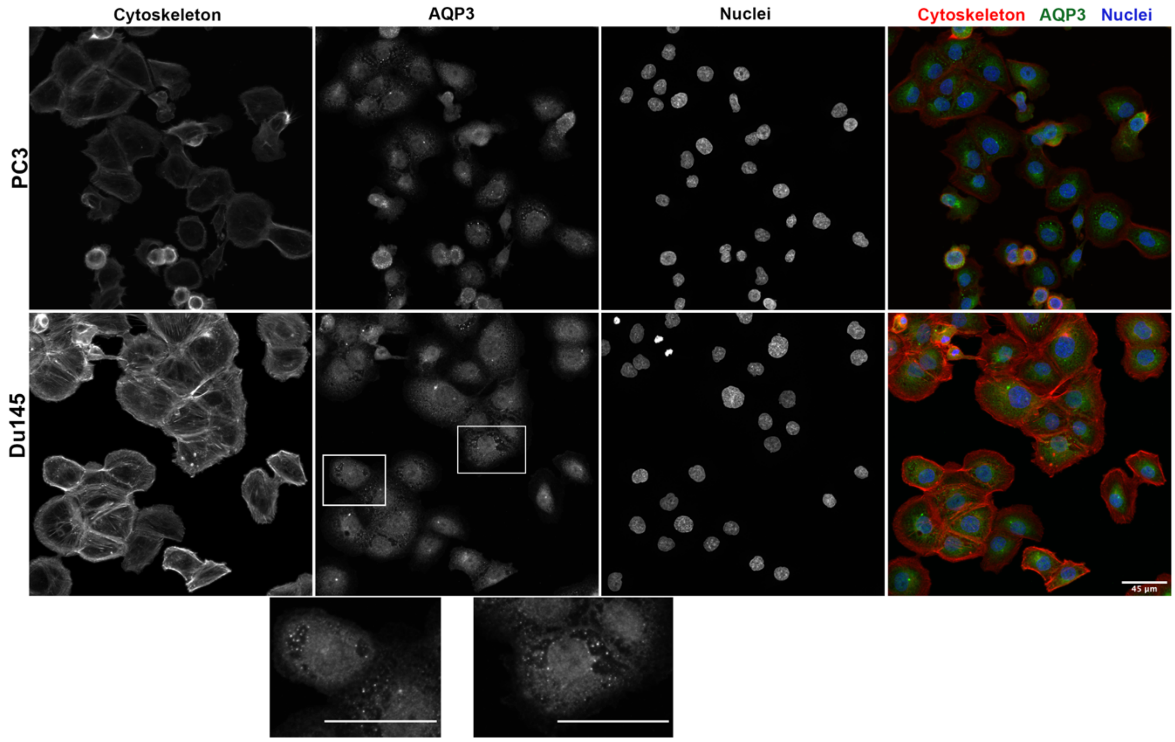

Figure S7: Maximum projection of Z-stack confocal microscopy images of Du145 cells under chronic hypoxia (8 weeks), in greyscale and merged channels. AQP3 is shown in green, while actin staining is shown in red and nucleus in blue. Scale bar represents 45 µm.

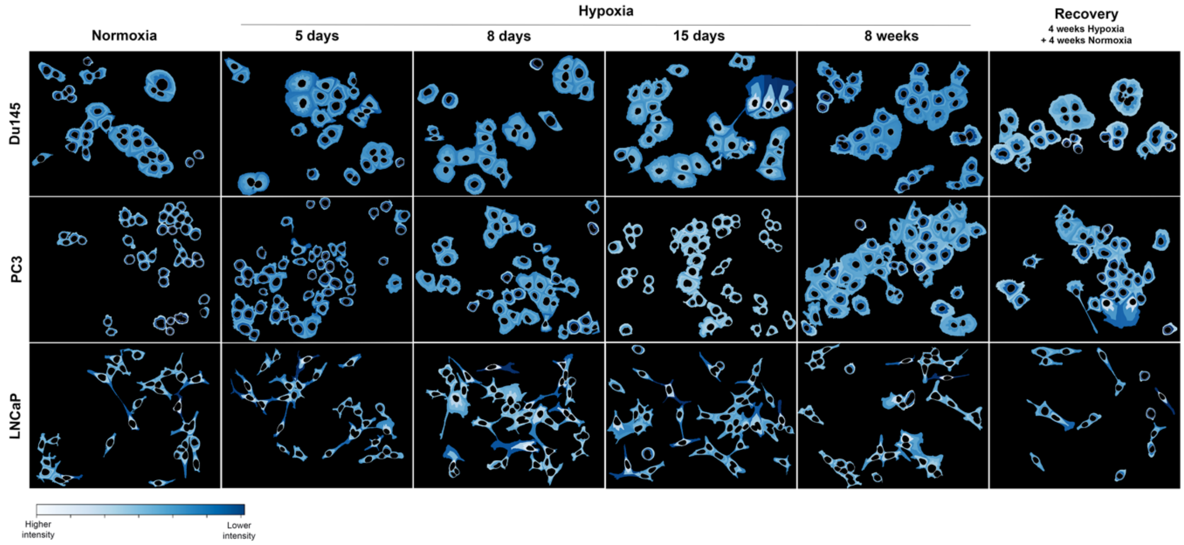

Figure S8: Fraction at distance graphical representation of AQP3 intensity in the cytoplasmic compartment, using 4 bins. Scale of intensity is normalised for each image individually, for its maximum and minimum intensity, with a lighter colour representing the highest intensity. Data from CellProfiler, from cells under acute hypoxia (days 5, 8 and 15), chronic hypoxia (8 weeks) and recovery (4 weeks hypoxia + 4 weeks normoxia).

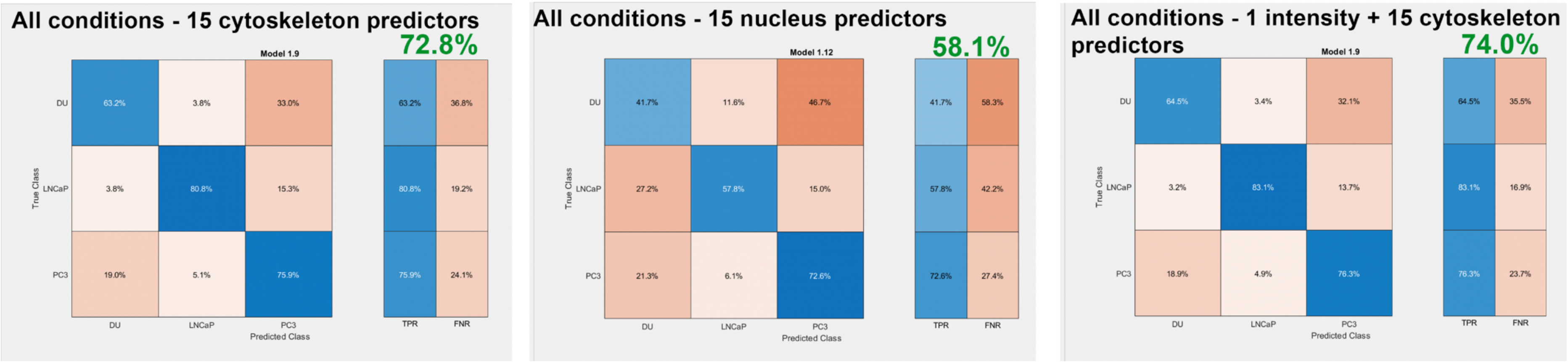

Figure S9: Confusion matrices summarising classification results for the three cell lines (DU145, LNCaP and PC3) when employing all six experimental conditions. The type and number of predictors are shown for each matrix. Overall accuracy is shown in green. TPR – True Positive Rates, FNR – False Negative Rates.

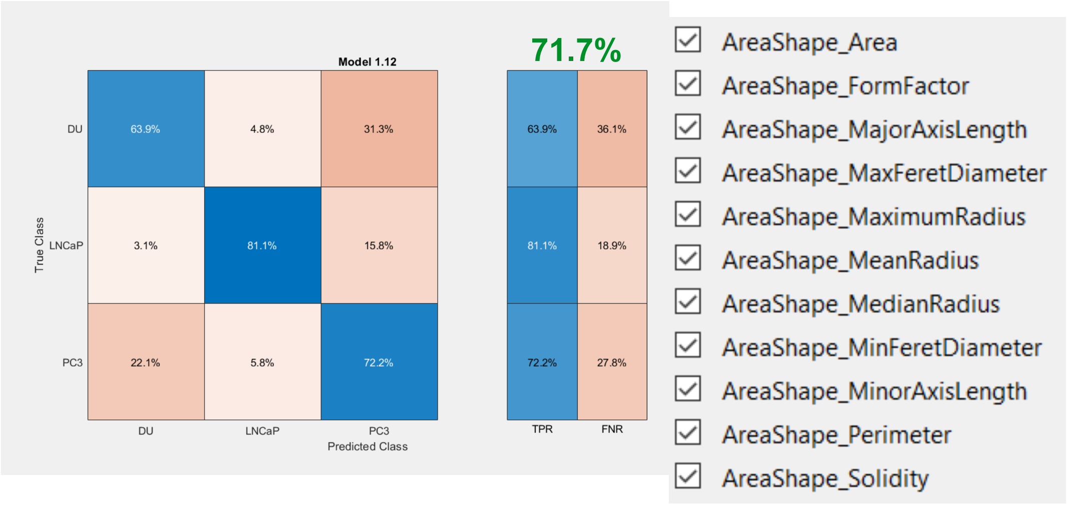

Figure S10: Confusion matrix for all conditions, classifying the different cell lines and using a subset of cytoskeleton-derived intensity predictors, as listed. Overall accuracy is shown in green. TPR – True Positive Rates, FNR – False Negative Rates.

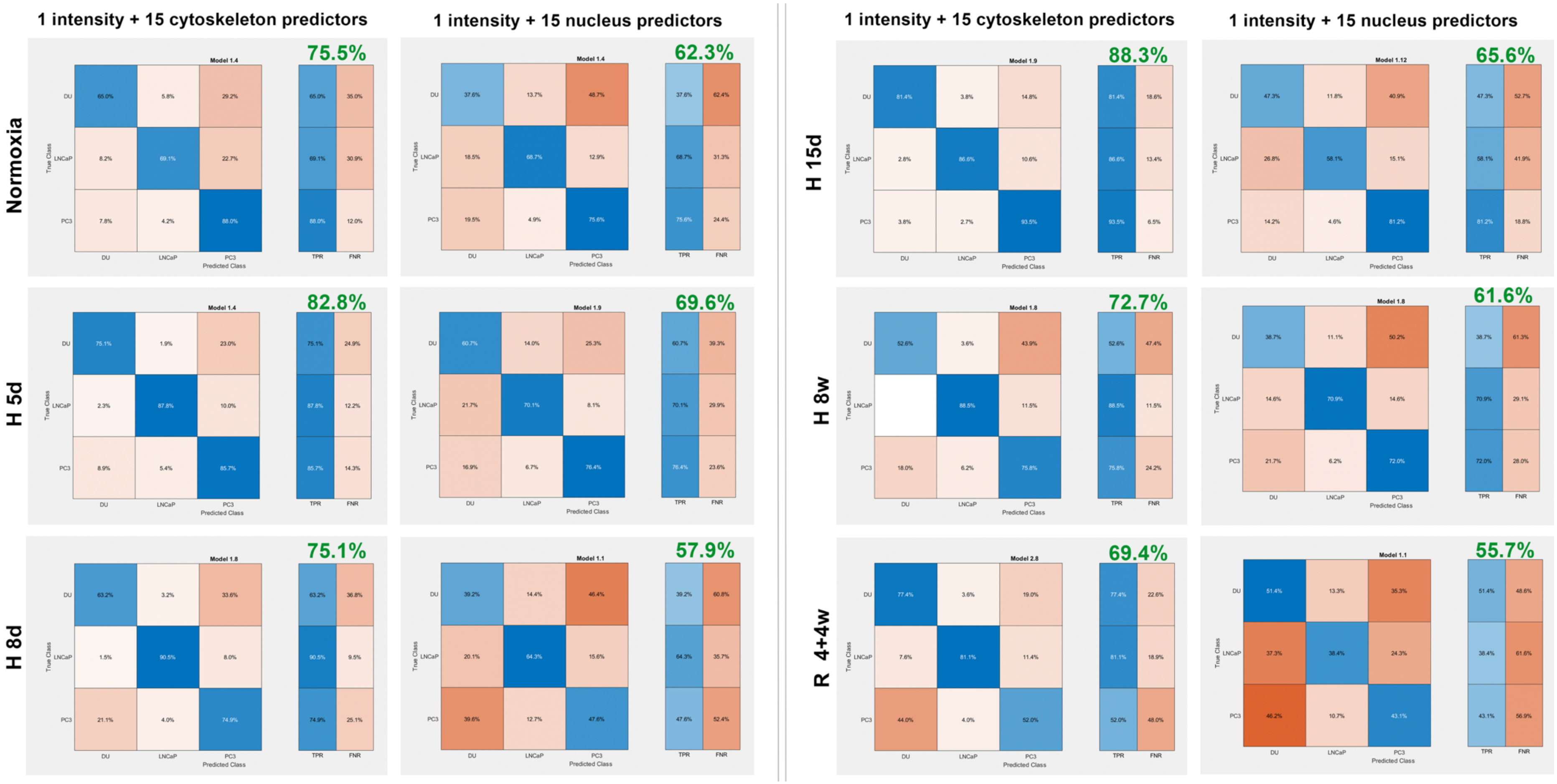

Figure S11: Confusion matrices for Normoxia, hypoxia (H) 5 days (5d) and 8 days (8d), 8 weeks (8w), and recovery (R 4+4w) used in the classification of DU145, LNCaP, PC3 cell lines. Classification using intensity/cytoskeleton or intensity/nucleus predictors, as shown. Overall accuracy for each case is shown in green. TPR – True Positive Rates, FNR – False Negative Rates.

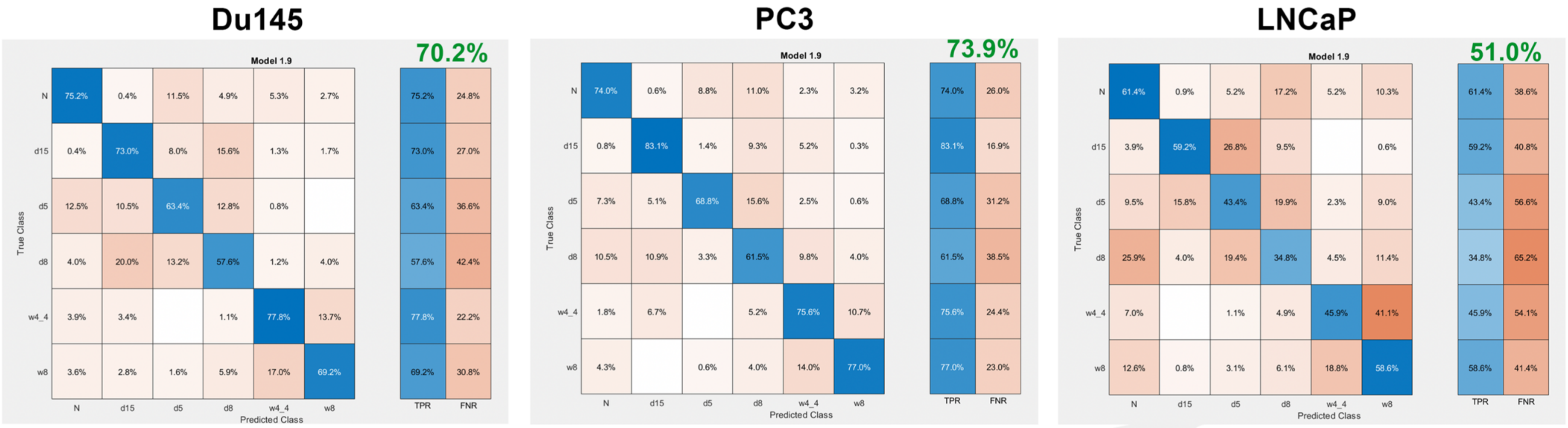

Figure S12: Confusion matrices for individual cell line, classifying the different conditions based on texture features predictor. Overall accuracy for each case is shown in green. TPR – True Positive Rates, FNR – False Negative Rates.

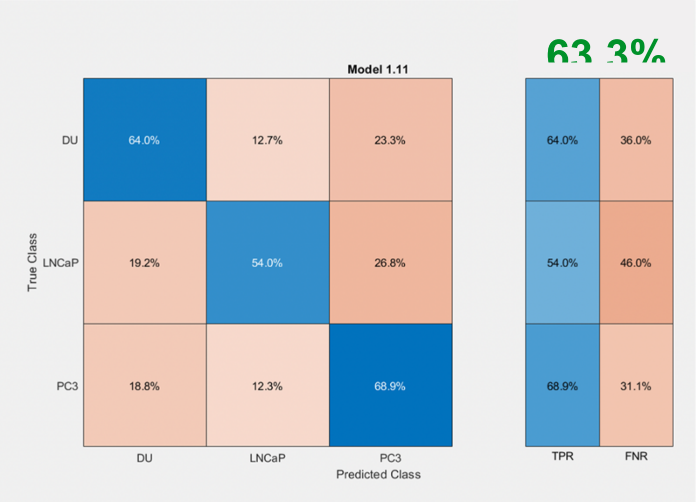

Figure S13: Confusion matrix for all conditions, classifying the different cell lines based on texture feature predictors. Overall accuracy is shown in green. TPR – True Positive Rates, FNR – False Negative Rates.

Publisher's Note: MDPI stays neutral with regard to jurisdictional claims in published maps and institutional affiliations.

© 2020 by the authors. Submitted for possible open access publication under the terms and conditions of the Creative Commons Attribution (CC BY) license (http://creativecommons.org/licenses/by/4.0/).
